# Development of a Wheelchair Skills Home Program for Older Adults Using a Participatory Action Design Approach

**DOI:** 10.1155/2014/172434

**Published:** 2014-09-04

**Authors:** Edward M. Giesbrecht, William C. Miller, Ian M. Mitchell, Roberta L. Woodgate

**Affiliations:** ^1^Department of Occupational Science and Occupational Therapy, University of British Columbia, T325-2211 Wesbrook Mall, Vancouver, BC, Canada V6T 2B5; ^2^Department of Computer Science, University of British Columbia, 2366 Main Mall, Vancouver, BC, Canada V6T 1Z4; ^3^Faculty of Nursing, University of Manitoba, 89 Curry Place, Winnipeg, MB, Canada R3T 2N2

## Abstract

Restricted mobility is the most common impairment among older adults and a manual wheelchair is often prescribed to address these limitations. However, limited access to rehabilitation services results in older adults typically receiving little or no mobility training when they receive a wheelchair. As an alternative and novel approach, we developed a therapist-monitored wheelchair skills home training program delivered via a computer tablet. To optimize efficacy and adherence, principles of self-efficacy and adult learning theory were foundational in the program design. A participatory action design approach was used to engage older adult wheelchair users, care providers, and prescribing clinicians in an iterative design and development process. A series of prototypes were fabricated and revised, based on feedback from eight stakeholder focus groups, until a final version was ready for evaluation in a clinical trial. Stakeholder contributions affirmed and enhanced the foundational theoretical principles and provided validation of the final product for the target population.

## 1. Introduction

Canada has a rapidly aging population [1] and it is estimated that, over the next 50 years, the proportion of Canadians over 65 years of age will double to more than 25% [2]. With advancing age, the risk of a disabling health condition increases and personal mobility is the most prevalent area of impairment among older adults [3]. A manual wheelchair (MWC) is often prescribed to address compromised mobility and function. In 2001, an estimated 81,000 Canadian older adults were wheelchair users [4]. The intent of providing assistive devices such as a MWC is to improve functional independence and diminish caregiver burden [5]. In practice, however, elder MWC users experience substantial restrictions in the performance of their activities of daily living [3] particularly in comparison with elders who do not use a mobility device [4, 6]. As a result, nearly 60% of older Canadian MWC users are dependent upon formal or informal care providers for basic mobility [4].

The international classification of functioning, disability, and health (ICF) provides a conceptual framework for describing factors that contribute to or impede participation [7], including participation among wheelchair users. For the individual, compromised body function and structure related to pain, strength, and endurance may be influential. Relevant contextual factors may be environmental. These include the built environment, such as pseudo-accessible [8] and inaccessible locations, and challenging terrain; the social environment, such as social attitudes and level of personal assistance; and assistive technology devices including wheelchairs that are low-quality and inappropriate or do not fit the user. Contextual factors may also be personal, such as age and confidence (self-efficacy) with wheelchair use. At the activity level, wheelchair mobility skill and proficiency have also been identified as significant contributors to participation [9–12]. Wheelchair skills have been amenable to improvement through training, particularly when delivered in a structured format. For example, there is considerable evidence supporting substantive benefits of the Wheelchair Skills Training Program [13] in a variety of populations and contexts [14–16].

Older adults typically receive little training when they obtain a wheelchair [17, 18], and whatever training they do receive tends to focus on functions related to hospital discharge (e.g., transferring from the wheelchair to bed or toilet). Insufficient training occurs for a variety of reasons, but primarily because therapists have limited time and must focus on pragmatic issues, and resources for follow-up services are restricted [19]. Coming in as an outpatient for multiple training sessions is costly—many visits, transportation, and inconvenience—and it does not provide training in the context of use (i.e., real-life obstacles). Providing training in the community would be desirable, but there are not resources to enable therapists to make multiple visits and provide training at home or in the community. This lack of comprehensive, context-appropriate training is particularly problematic because older adults require more time and training to acquire new motor skills due to age-related changes in motor, sensory, and cognitive function [20, 21].

To address this problem, we set out to develop a monitored home program for wheelchair skills training. Delivering rehabilitation training as a monitored or self-managed home program among older adults has been effective for a wide variety of outcomes including strengthening [22], physical activity [23], self-care [24, 25], and exercise [26, 27]. Home programs are advantageous because they allow privacy for the user, occur in a familiar and real-life context, can be conveniently integrated into the users schedule, and do not require the time, effort, and expense of travel to another location [23]. This approach would be economically viable, allow time for more practice, and facilitate training in the context of using authentic obstacles.

For rehabilitation home programs targeting motor skills in older adults, maximizing training frequency and practice in the natural context of use are essential elements. A 2010 Cochrane Review identified several factors related to adherence in exercise interventions. Programs that incorporated social cognitive theory (i.e., self-efficacy strategies), were clinician-monitored, and increasingly graded the complexity of training activities were more successful improving participants' adherence, frequency and duration of exercise [28]. Education is central to rehabilitation and training should utilize strategies from Andragogy (adult learning) [29, 30] as active ingredients to promote program adherence and successful skill acquisition with older adults [31].

With advances in affordability, size, portability, accessibility, and user-interface simplicity, computer-related devices are becoming increasingly useful for rehabilitation interventions. Computer and popular gaming systems (e.g., Nintendo Wii) have shown promising results in rehabilitation by casting therapy in a more engaging context. More recently, their use in rehabilitation among older adults has also been explored. For example, Aarhus et al. [32] provided physical activity training in a Danish nursing home using a commercial gaming system with a participation rate over 90% and found improvements in physical function, increased motivation and tolerance for activity, and trends towards improvement in fitness. Imam et al.  [33] demonstrated improved mobility outcomes among individuals with lower limb amputation (age range: 45–59 years) using the Nintendo Wii to augment usual therapy and reported high rates of adherence and enjoyment with the program. Creating an interesting interface, such as the use of games, is positively associated with older adults' intention to use computer-related technologies [32].

*Purpose.* The purpose of this project was to develop a prototype Wheelchair Skills Training Program that could be delivered as a home program using a computer tablet, entitled Enhancing Participation in the Community by Improving Wheelchair Skills or EPIC Wheels. This involved the development of specific program content as well as a system of delivery. The intent was for content to include evidence-based skills relevant to novice older adult MWC users, incorporate Andragogical educational strategies, promote self-efficacy, and be delivered in an engaging and accessible format. The specific objectives were to

1. engage older adult MWC users in the research and development process,
2. incorporate stakeholder input through the design and evaluation phase,
3. produce a prototype intervention program for proof of concept.

## 2. Materials and Methods

There is an emerging consensus in the field of assistive technology that consumer involvement* during* the process of intervention development is crucial [34–36]. This is particularly true with older adults to ensure that a technology “solution” itself does not induce more problems than it resolves [36]. An additional benefit of involving older adults is the “Design for All” tenet that assistive technology interventions that work well for the elderly are also likely to work better for consumers generally  [36].

**Figure 1:**
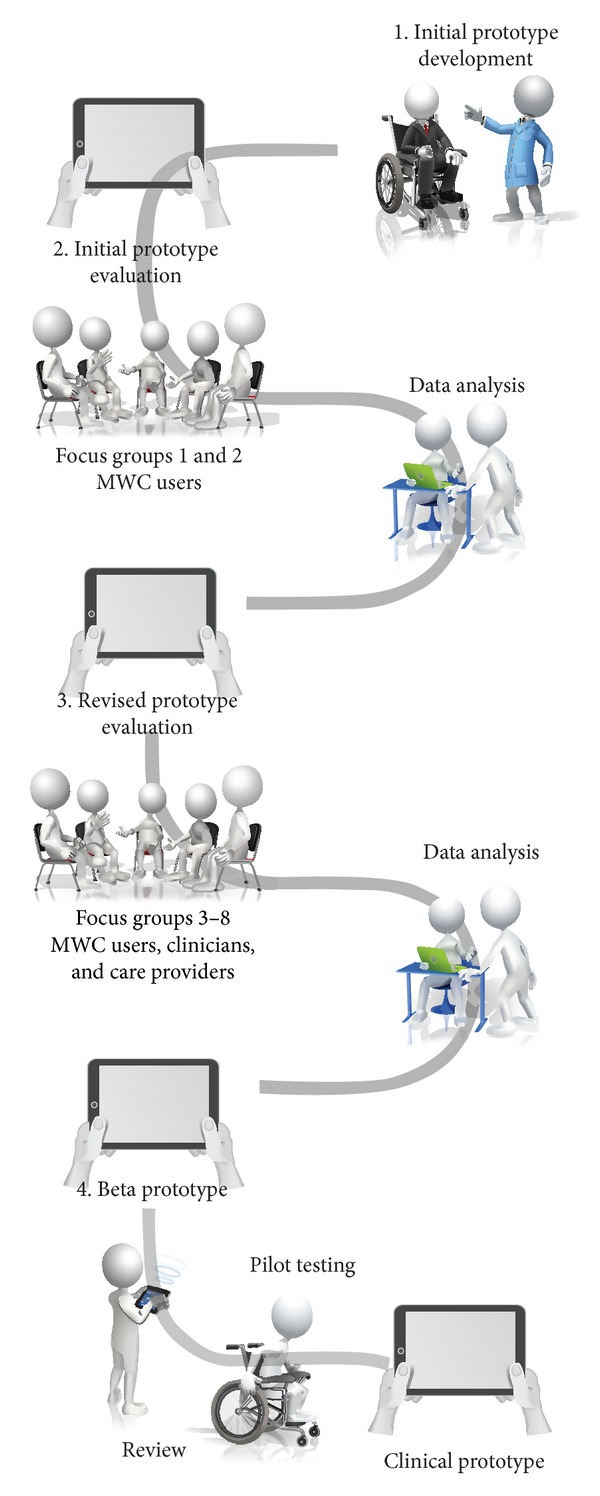
Phased study design.

We employed participatory action design (PAD), which is an approach to innovation development that places high value in the on-going involvement of intended users during design and evaluation elements [37–39]. Using a PAD framework, stakeholder evaluation and feedback were incorporated into the development stages of program content and delivery through the use of focus groups (see Figure 1). Focus groups were used to capitalize on participant interaction to elicit needs and preferences, personal experiences, and exploratory solutions “outside of the box” [36] and have proved effective in other comparable participatory rehabilitation intervention studies [35–37, 40]. Including a qualitative approach ensured that learning strategies were relevant for older adults, practice activities were age-appropriate and achievable, potential for user motivation and adherence was maximized, and the product design considered the technological accessibility needs of an aging population. The study followed an iterative pattern where issues of importance are identified, prototypes are developed and refined, and the results are evaluated for utility (see Figure 1).

A total of eight focus groups were conducted in two cities: Winnipeg and Vancouver. These locations provided diversity in culture, weather, geography, and degree of wheelchair accessibility and would also serve as research sites for a subsequent clinical trial of the program. Finally, two older adult MWC users (one experienced user and one novice user) pilot tested the prototype using a research protocol intended for the subsequent clinical trial. Details of the Android software development have been published separately [41]. All participants provided consent and approval from the university affiliated Research Ethics Board at each site was obtained prior to conducting this study.

### 2.1. Participants

Three stakeholder groups in each city were included: experienced MWC users aged 60 and over, care providers of older adult MWC users, and clinicians who prescribed wheelchairs and/or provided wheelchair training for older adults. MWC users were the primary stakeholder group as we were most interested in their perception of the program content and delivery, since adherence to a home program is critical to effectiveness [28]. The user groups (*n* = 10) each participated in two focus groups (at different points in the program development), while care provider and clinician groups each attended one focus group; separate focus groups were conducted with each stakeholder group in each city. While the target population for the training program is* novice* users, we chose to use* experienced* users for several reasons. First, we anticipated that their availability and potential for attendance would be greater since they would have either acquired mobility skills or developed compensatory strategies over time. It was also more likely that whatever impairment precipitated their acquisition of a wheelchair would have stabilized sufficiently that they would be able to schedule and attend two focus groups. Second, novice users often experience a transitional period of emotional and social adjustment, and engagement in a research study might prove challenging [42, 43]. We reasoned that novice users would have a more limited experience and perspective to know what it was they* did not know* and the full scope of environmental situations that posed the greatest barriers to mobility and participation. Conversely, experienced users, while somewhat distanced from the “experience” of early adjustment to wheelchair use, would have a more comprehensive understanding of the scope of environmental barriers and could reflect on which barriers were most problematic and which mobility skills had been most important or influential in addressing participation restriction.

The MWC user and care provider participants were recruited using email and postal invitations, public advertisement, and word-of-mouth. MWC users were at least 60 years of age, were living in the community, had used a MWC as their primary means of mobility for at least one year, and have sufficient cognition and English language skills to engage in the focus group process. Care providers were individuals (e.g., spouse, relative, or caregiver) who assisted or accompanied a MWC user at least 60 years of age while using their wheelchair inside and outside of the home. For the clinician group, occupational and physical therapists at the largest rehabilitation hospital in each city who supervise or provide clinical services (e.g., prescribe a wheelchair or provide wheelchair mobility training) to individuals 60 years of age or older were invited to a lunch-hour focus group. Advertising posters and brochures were distributed to therapists at each site and local rehabilitation managers distributed invitations to their staff via email. All participants provided informed consent prior to participating in this study.

A total of 10 MWC users participated in the focus groups. At the Vancouver site (*n* = 6), one individual was not able to attend the second focus group due to weather conditions. At the Winnipeg site (*n* = 4), two participants attended both focus groups while two attended only one focus group. The mean age of MWC users was 66.8 years (range 55–83 years) and had used a wheelchair for a mean of 31.9 years (range 4–60 years). Among the care providers, there were 2 participants at each site (*n* = 4) and all were female. At the Winnipeg site, Jamie was in her 30s and worked in an intentional community home where she was a caregiver for a variety of individuals with a disability, some of who were older adult wheelchair users. Felicia was in her 60s and assisted her husband who was in his 70s and used both a manual and power wheelchair. In Vancouver, Patricia assisted her husband and Bertha provided care for her daughter; in both cases, the care recipient had been a participant in the MWC user focus groups as well. A total of 20 clinicians participated in focus groups between the Winnipeg (*n* = 9) and Vancouver (*n* = 11) sites.

### 2.2. Data Collection and Analyses

The collection and analyses of the focus group data were central to the program development and revision process. One of the coinvestigators (EG), who had experience in focus group facilitation and expertise in the content area [44–46], cofacilitated all focus groups with a research assistant. All sessions followed a similar format: a brief introduction and audiovisual presentation of the study background, purpose, and design; interactive discussion using a semistructured guide designed for each respective stakeholder group; and opportunity to interact with the training program prototype and provide feedback. The discussion guide included background questions around previous wheelchair training experience and barriers to effective use, motivation for practicing and improving skills, and effective learning strategies and techniques. Discussion occurred prior to presentation of the tablet device and proposed content/features to avoid restricting the scope and breadth of participant feedback. Following the prototype presentation, discussion questions shifted to participants impressions of the program content and delivery; perceptions about appropriateness, engagement, and motivation; and suggestions for revision, addition, or elimination. Each session was audio-recorded and transcribed verbatim by the research assistant, and both facilitators kept field notes of their experience. A second research assistant verified the accuracy of the transcripts against the audio recordings before personal identifiers were removed. Portions of the user and care provider sessions were video-recorded so the investigators could observe participant interactions with the computer tablet.

A* directed content analysis *approach [47]was used with data from each focus group. The initial coding scheme was informed by concepts and themes from Andragogy and Social Cognitive theory, and Edward M. Giesbrecht parsed the content assigning codes to each discrete element, with emergent codes being integrated after each subsequent focus group. The research team met regularly to discuss analysis and coinvestigators WCM and RLW reviewed coding. Any discrepancies were discussed until consensus was reached. An audit trail of the research and analysis process, including all coding procedures, was documented by Edward M. Giesbrecht [48]. Participant collaborators also engaged in this process through debriefing and member checking at the second focus group.

Data analyses and intervention development took place concurrently as some participant feedback was self-evident and easily implemented (e.g., size of icons, number of menus) while other revisions required more in-depth analysis (e.g., relevance of activities and appropriateness of training approach). This iterative feedback/revision process began with an* initial prototype* followed by the development of a* revised* prototype after the first set of focus groups and a* beta* prototype after the second set, culminating in a* clinical prototype* following the final review and pilot-testing phase (see Figure 1).

## 3. Results

### 3.1. Initial Prototype Development

A variety of evidence-based resources were used to create the initial content outline, including the Wheelchair Skills Training Program, which is a comprehensive structured curriculum available online [13]. Initially four categories of content were created: safety, wheelchair components, body position, and mobility skills. The mobility skills were structured sequentially and grouped into natural categories, based on underlying prerequisite skills and increasing performance complexity or difficulty. A script was created with the intent of delivering content through a series of short video presentations. Training activities were developed for each curriculum component. To facilitate tablet presentation during focus groups, a “mock” program framework was created with an interactive menu. Several preliminary video segments were integrated (e.g., safety, demonstration of one skill, and sample training activity) for demonstration, but links and proposed features (e.g., the trainee-trainer voicemail function) were nonfunctional placeholders. A storyboard was used to outline the desired sequence and configuration of content. One of the authors (Ian M. Mitchell) oversaw development of the initial software and tablet user-interface.

The development team met regularly in person, via telephone, and by email to discuss design issues, curriculum content, and program delivery. Following each focus group data analysis phase, stakeholder feedback was presented to the team and further redevelopment work was undertaken. The study team used a consensus process to decide which revisions and additions proposed by focus group participants would be incorporated based on consistency with the conceptual framework. Where recommendations were technically and economically feasible, we tended to be inclusive given the fact that the subsequent feasibility trial would enable additional exploration of which features were most beneficial. The following sections outline stakeholder response and subsequent revision in greater detail.

### 3.2. Initial Prototype Evaluation: MWC User Response

Participant responses fell into three major themes:* challenges to wheelchair use*,* optimizing strategies for learning skills*, and* critiques of the tablet device*. The input of the MWC user group provided confirmation for elements of the EPIC Wheels program content and strategies for delivering training and also resulted in several changes to the initial program prototype.

#### 3.2.1. Content: Challenges to Wheelchair Use

The focus group discussion guide explicitly intended to elicit from experienced older MWC users the types of environments and activities that were most problematic and the skills that were most beneficial to enhancing participation. MWC users indicated that maneuvering in confined spaces indoors was difficult, particularly doorways, around furniture in small rooms, and negotiating tight corners. Skills such as tight, accurate turns and alternating forward and backwards movements were critical in these situations. Small elevation changes were also noted, such as doorway thresholds and sidewalk cracks or heaves, which can catch the front casters and initiate a forward tip. Soft or accommodating surfaces, such as grass, carpet, and gravel, were particularly difficult for older users with compromised strength. Participants reported that ramps and inclines required both effort and control, coordinating hand movements to prevent rollback during ascent and limiting speed during descent. Curbs and steps were identified as substantial barriers to ascend independently and daunting to descend due to the risk of a forward tip.

Participants also identified awareness of how wheelchair components operate as important to efficient use of the wheelchair. Specifically, operating the wheel locks (brakes) and positioning of the front casters were important knowledge-based components of wheelchair operation. In addition, participants highlighted the relevance of their position within the wheelchair and the impact on operation and responsiveness. For example, leaning forwards or backwards alters the weight distribution between the front and rear wheels, increasing or decreasing wheelchair stability.

#### 3.2.2. Training: Optimizing Strategies for Learning Skills

The older adult MWC users spoke of the importance of a visual demonstration of each skill. Participants preferred “seeing” the task requirements before attempting performance. For example, getting over a doorway threshold could be broken down into positioning casters upon approach, shifting weight backwards, popping the casters over with a quick push, and forward weight shift while propelling the drive wheels over the threshold. Furthermore, demonstration by an older adult peer was deemed to be particularly helpful. Participants cautioned that seeing only “correct” performance was not sufficient. As Ted states: “*So do not always show the successful way … show us a way you could go wrong too,*” suggesting training should also include implications of incorrect performance particularly related to safety, including a demonstration. In addition to authentic demonstration models, participants advocated that training should occur in real environments using actual obstacles. In particular, training should occur in the home or community, where the obstacles encountered were truly representative of life situations rather than ones that might be constructed in a clinical setting.

In learning mobility skills, participants stated that success was important to bolster enthusiasm and confidence and that training activities should begin with simple and achievable skills before progressing to more difficult ones. The transition between activities should be graded and the initial speed of performance should be slower to ensure safety. Participants recommended an individualized approach focusing on skills relevant to the specific user, with the trainee having some control over which skills are practiced. Providing a rationale for using each specific skill was stressed. For example, the skill should be presented in the context of a particular situation and explain how acquisition of the skill will improve performance or reduce the risk of injury when performing a relevant activity.

Participants indicated that training activities needed to be engaging and interactive to promote adherence and overcome initial hesitation that might result from fear, low confidence, or apathy. The importance of the relationship between trainer and trainee was noted, identifying that personal contact, individualized evaluation, and feedback would contribute to greater motivation.

#### 3.2.3. Interface: Critiques of the Tablet Device

Participants were impressed with the tablet device as a potential training device. In particular, the portability for use in a community context and the capacity for visual demonstration of individual skills and skill components were highlighted. Participants noted the tablet's built-in capacity for video recording trainee performance had great potential for learning. Concern was raised around the potential for the tablet to be lost, misplaced, or stolen given its small size; ironically, one participant returned to the meeting room shortly after the focus group had finished to locate and retrieve their cell phone.

During the demonstration, we indicated that the training content on the tablet would be delivered using the Internet. Participants expressed apprehension about this dependence on Internet connectivity and what might happen if trainees were without Internet access. Finally, there was considerable discussion around receptivity and capacity of older adults to use the tablet technology. In particular, some participants wondered whether older adults would have the cognitive and attention ability to learn to use the tablet in addition to learning wheelchair mobility skills. This discussion generally reflected participants' perceptions about other older adults and, in particular, those in their late 70s and 80s. All of the focus group participants were over 60 and felt this technology would not be particularly challenging or intimidating for them to use; however, some felt that this might not be the case for others older than they were:

*“My mother just got an ipad and let me tell you I'm spending more time with my mother (laughs) … even her touching the screen to select things is a real challenge … she often gets totally discombobulated … but for someone like me … it's second nature” (Louise, 61, Spina Bifida)*

* “The tablets are neat, but … I guess I've got this sort of idea or intuitive sense that people are going to be older … closer to 70… even for me I'm familiar with that kind of stuff but … [for others] it just seems to be easier just to [use a DVD]” (Michelle, 63, Polio).*

In particular, participants wondered whether some older adults would have difficulty navigating through multiple menus and icon options and become “lost.”

*Revisions to Initial Program Prototype*. The participants' reporting on common challenges to wheelchair mobility provided confirmation for the content areas proposed in our initial prototype. The specific skills related to addressing environmental barriers, such as propelling straight, turning, and popping casters, were all contained in the training curriculum. The initial prototype had, by design, only a limited repertoire of video content as we anticipated substantial revision. In response to the challenges noted with negotiating small, crowded spaces, we expanded the content related to turning and maneuvering skills. For example, wheelchair casters have an off-center pivot, swinging into a trailing position when initially moving forwards and a leading position when reversing. During these transitions, the wheelchair has a tendency to veer towards one side. A training segment was added specifically addressing this response and how to best control caster swivel. Content was expanded to include explanation of the “mechanics” of turning a wheelchair and broken down into small progressive segments including stationary turns, stopping and turning, moving turns, spin turns, and backward turns. Manipulating body position within the wheelchair to improve stability, safety, and responsiveness of the wheelchair became a separate content area early in the program, as it is a prerequisite for many advanced mobility skills. Additional content was also added related to safety, based on participants' feedback. A separate section identifying equipment (i.e., antitippers, spotter's strap, and gloves) was added as well as educational information regarding tips and falls, spotting/supervision, feedback during training, and injury prevention.

For convenience and expediency, the initial prototype included video demonstrations featuring the first author. In the subsequent prototype, two individuals over 65 years of age (one male and one female) were recruited to model skill performance and training activities. As suggested in the focus groups, a number of skills were enacted with common* errors* to illustrate how and why unsuccessful performance might occur. These included both naturally occurring and contrived errors. Naturally, occurring errors emerged when models were initially unsuccessful attempting a skill and proved useful in demonstrating how to correct mistakes as the models adjusted their approach. Contrived errors were useful as a “3 bears” approach to learning (i.e., demonstrating what happens if you turn too soon, too late, and “just right”) and addressed the recommendation to link potential consequences with skill performance.

To address concerns about network connectivity, the system was modified so that it could operate with only sporadic Internet access. In particular, video viewing and training could take place with or without being connected; however, brief connections were still occasionally required so that data and messages could be exchanged with the monitoring trainer.

### 3.3. Revised Prototype Evaluation

For the second round of focus groups, which involved MWC users, care providers, and clinicians, their responses were again categorized into three general themes:* challenges to wheelchair use*,* optimizing strategies for learning skills*, and* critiques of the tablet training device*. There was overlap between the stakeholder group feedback as well as unique contributions from the three perspectives.

#### 3.3.1. Content: Challenges to Wheelchair Use

With the revised prototype, most of the situations and environments stakeholders identified (and the requisite skills for performance) were contained within the training program content. The clinicians highlighted the challenges of uneven, undulating, and irregular surfaces for older adult MWC users, which were particularly taxing on their endurance. This included working against gravity pushing both up- and downhill and lost momentum when stopping to overcome small gaps or changes in elevation. There was general consensus that performing a sustained wheelie was not an essential or even high priority skill for this user group, but the* transient wheelie* (i.e., popping the casters) was unquestionably a useful and productive strategy to learn.

The care provider groups identified functional upper extremity activities as problematic. This included reaching for objects on the floor and at height, such as operating a ticket kiosk in a public transit station. Using doors was also noted as difficult because it involved manipulating the wheelchair and the door simultaneously and can be compounded by mechanical closers. One MWC user group proposed inclusion of a section on carrying objects, a skill not previously identified specifically in the literature. Since propulsion is often a bilateral activity, transporting an object (such as a cup of hot coffee) is particularly problematic.

Both the MWC user and clinician groups noted the particular challenges of propelling on snow and ice; this was true at both sites, despite the substantial differences in climate between the cities (mean days of snowfall: Winnipeg 53, Vancouver 11; mean depth of snow between December and March: Winnipeg 15 cm, Vancouver 0 cm). Snow can be particularly soft and conforming to the casters, causing them to become buried and the wheelchair to “snowplow” or stop suddenly, causing a risk of forward tipping. In addition, low friction reduces traction at the rear wheels, resulting in one or both wheels spinning.

#### 3.3.2. Training: Optimizing Strategies for Learning Skills

The clinician groups identified that in training older adults, memory for new learning is often a challenge and needs to be addressed through increased repetition and breaking skills into smaller steps. While they spoke positively about the content of the training videos, both the MWC user and clinician groups indicated the importance of using lay terminology and avoiding excessive technical jargon. At the same time, the MWC users suggested value in using accurate terminology for the wheelchair components to ensure consistency and clarity throughout the learning process. Consistent with the MWC users in round one, the clinicians identified value in describing the benefit of each skill for trainees but also suggested this was important for family and care providers to secure their support in the training process. The care providers wondered whether there might be a benefit to trainees being able to navigate through individual videos to rewind or fast-forward depending upon their learning needs and desires. They affirmed the use of games and interactive activities to engage the trainee in practice, such as the “roller coaster” activity that requires trainees to lean backwards, forwards, and sideways in their wheelchair as the roller coaster car ascends, descends, and turns along the tracks. Care providers also highlighted the need for flexibility to select individual skills and activities, rather than having to follow a prescribed sequence.

The clinician groups suggested that the training program should include not only skills for independent mobility targeting the user but also skills and techniques for care providers to assist older MWC user when independent mobility is not feasible. This was particularly true for skills that might not be reasonably achieved independently, such as managing steps or curbs safely.

#### 3.3.3. Interface: Critiques of the Tablet Device

The clinicians commented that the instruction and demonstration videos were not all of one uniform size and suggested greater consistency and, more importantly, maximizing the size of the video image. Care providers commented that the tablet surface has a significant glare which compromised viewing, particularly when positioned on an angle. The buttons were described as being adequate but somewhat small, and the text was hard to read for some. Likewise, the volume was described as adequate but could potentially be problematic for trainees with compromised hearing. Both the user and care provider groups wondered how the tablet might be positioned and supported during training activities and the risk of it falling to the floor and being damaged.

*Revisions to Second Program Prototype*. In response to the stakeholder feedback, several additional content areas were introduced. Within the training section related to soft surfaces, we added instruction and video footage on propelling over snow and ice. We also incorporated content specific to care provider (assisted) mobility skills such as getting up and down curbs, steps, and ramps and using the tipping bar to get over small obstacles. Managing doors (with and without closers) and strategies for carrying objects were incorporated as distinct sections.

Several changes were made to the tablet display and user-interface. Video clips were configured to display in the same size configuration. Navigation buttons (e.g., play, pause, and stop) were relocated from below the video image (horizontally) to the right of the image (vertically) to maximize image height and permit a widescreen display. The vertical orientation also permitted an increase in the size of the buttons for easier targeting [49], along with decreasing the amount of text and increasing font size to address visual acuity changes with aging.

We proposed a training schedule of 30 minutes per day (1-2 sessions per day for 15–30 minutes each) at least 5 days per week totaling a minimum of 150 minutes per week. These guidelines are based on the National Blueprint consensus document on promoting physical activity for adults over 50 years, which advocates that lifestyle- or endurance-related activity of moderate intensity should be undertaken for at least 30 minutes (in bouts of at least 10 minutes) 5–7 days per week [50]. All 3 stakeholder groups affirmed this schedule as reasonable and appropriate for the target population.

To address the potential issues with users becoming “lost” during program navigation, we developed 2 strategies—pretraining and reference material. The EPIC Wheels program incorporates two 1 : 1 training sessions with an experienced trainer. In practice, these sessions might occur shortly after an older adult obtains their wheelchair. As part of the initial evaluation and training session, we included a 30-minute interactive orientation to the tablet for the user and care provider. Trainees also receive a handbook that provides instructions for tablet navigation, including screenshots for visual assistance. For simplicity, menus were configured to have 3–8 options related by content area, limiting clutter, and distraction without requiring an excessive number of embedded submenus  [51]. We also addressed potential audio issues by including headphones, as augmented audio output increases usability for older adult users of touchscreen technology  [51].

**Figure 2:**
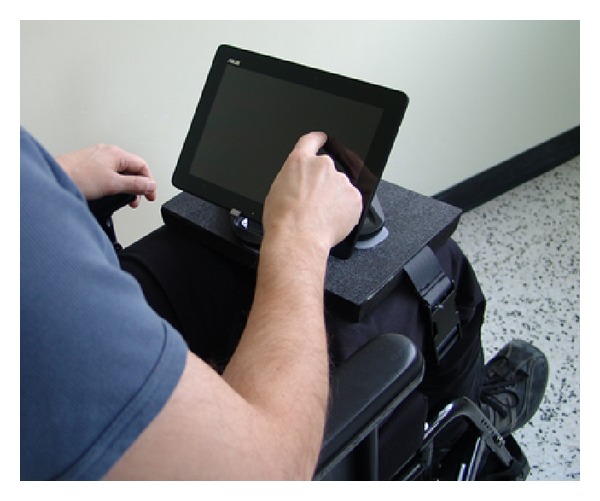
Tablet mounting platform for wheelchair use.

The first author (Edward M. Giesbrecht) and a rehabilitation engineer created a lap-mounted support to enable viewing and practice without risk of loss of or damage to the tablet while trainees sit in their wheelchair. A nylon strap and buckle were integrated into a rigid platform with a neoprene foam base, upon which a commercial tablet holder (Cyber Acoustics IS-4000 Universal Tablet Stand, Vancouver, WA) was mounted using hook and loop fasteners (see Figure 2). The tablet could be used in chair or easily removed and placed on a table or other surfaces if desired. A training “kit” was created using common household objects (e.g., boxes, balls, balloons, etc.) at a total cost of less than $20 and could fit in a grocery bag. A kit would be provided to trainees to support all of the tablet-based training activities.

### 3.4. Beta Prototype: Review and Pilot Testing

Following revision, we met individually with one of the MWC users and one clinician for a final review of the beta prototype. Both reviewers provided confirmation of the scope and presentation of the training content and usability of the user interface, and no substantive revisions were required. In particular, the MWC user was pleased with the tablet holder, indicating it was easy to don and doff in the wheelchair and provided a good viewing location with adequate adjustability. Subsequently, we conducted pilot testing of the EPIC Wheels program in preparation for a randomized controlled trial. A primary intent was to evaluate the robustness and feasibility of the EPIC Wheels home program and supporting technology. We selected two older adults with diverse wheelchair backgrounds who had no previous involvement in the study. One participant was very experienced, having used a MWC for over 30 years following a spinal cord injury, including participation as a wheelchair athlete in earlier years. The second participant had recently transitioned to MWC use (<6 months) following an above-knee amputation. Participants with diverse wheelchair experience were intentionally selected to obtain perspective from individuals new to MWC use (to determine the acceptability and potential benefit of the EPIC Wheels program) and proficient users (to ensure comprehensiveness and accuracy of the program content). Because the feedback from the reviewers and pilot participants was obtained during tablet use, it was not audio-recorded and transcribed as with the focus groups. Observations and concerns were recorded by the first author and consolidated with the previously obtained data.

The EPIC Wheels intervention was 4 weeks in length. Participants attended a 1 : 1 session with their trainer (an occupational therapist with wheelchair skills expertise) at the beginning of weeks 1 and 3 and trained at home the remaining days using the tablet device, with the trainer making follow-up calls at the end of the first and third weeks. Participants can use the voicemail feature as a built-in function of the EPIC Wheels program to exchange messages with their trainer, if desired; trainers can respond via their website interface. Because many potential trainees will not have broadband Internet access at home and the Android tablets used in EPIC Wheels are Wi-Fi only, trainees were also given a mobile cellular hotspot device. The tablet can connect through this device to the Internet to upload training data and exchange voicemail messages with the trainer.

After the study period, participants completed a program evaluation questionnaire and provided informal feedback on their experience. As anticipated, the experienced participant indicated he had not learned any new skills but had already been fully proficient with all desired mobility skills for many years. He indicated he would have appreciated such a program when he first obtained his wheelchair and felt there were no important skills or components missing from the program. He commented that the training activities were engaging and fun, although he modified some to increase the challenge because of his level of proficiency. The novice user also found the program comprehensive and identified a substantial benefit, not only due to his capacity for wheelchair mobility but also due to his ability to participate in meaningful activities of daily living. In particular, he identified improvement in turning (related to mobility in tight spaces) and learning to “pop the casters” enabled him to traverse small obstacles like doorway thresholds and propel over soft surfaces like carpet and snow. The lack of large open spaces and hard floor surfaces in his condominium presented some challenges with training activities. The experienced user did not use the voicemail feature, while the novice user had several voicemail exchanges with the trainer. Intermittent connectivity issues with the hotspot device resulted in some data uploading delays and this participant reverted to telephone calls rather than voicemail.

### 3.5. Clinical Prototype

**Figure 3:**
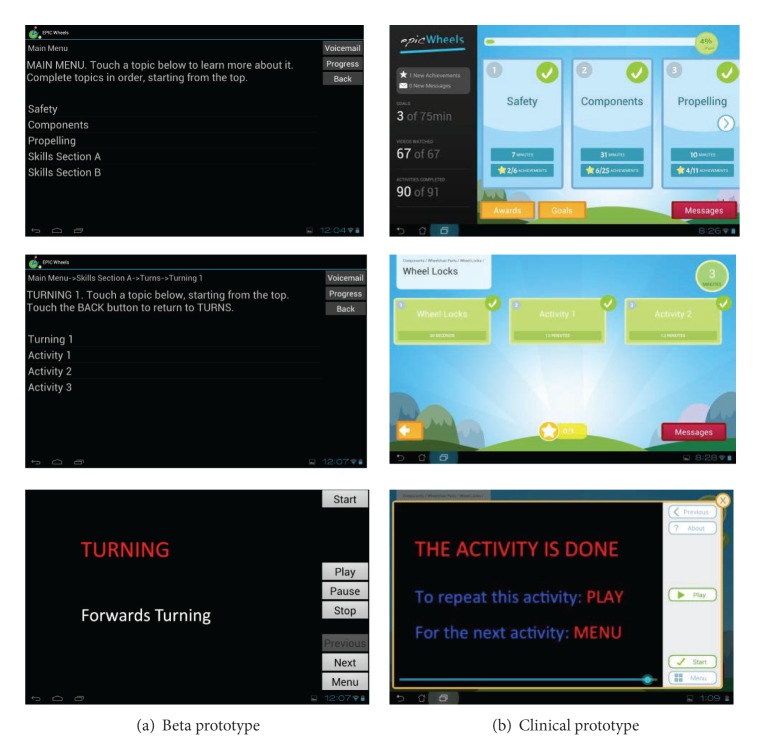
EPIC Wheels home screen and sample submenu.

Several additional changes were made to the clinical prototype, which would be used in a subsequent randomized controlled trial [52].

(1)* Upgrade to the User-Interface Software Program*. The training program was given a more bright and appealing appearance, similar to a commercial software application. The EPIC Wheels program automatically loads upon powering up or waking up the tablet. To increase ease of use and minimize distraction, there are no other applications or features visible. The home screen provides information on the number of minutes spent viewing instructional videos, minutes spent on training activities, and a graphic with weekly progress compared against the goal of 150 minutes (see Figure 3). Videos are accessed through five submenus arranged by content, with blue icons indicating a further embedded menu and green icons indicating that a video will play. A legend at the top of the screen indicates current location within the program menus. All videos display a slider bar with time played/remaining as well as a menu with buttons (previous/next video, play/pause, and exit/back). A stopwatch-like timer with a start/stop button allows trainees to record the amount of time spent on self-training activities. Once a training video or activity is accessed a “check mark” appears on the corresponding button, while a “star” is awarded after completion. To increase motivation, a series of “awards” are provided after completing an increasing number of training activities; trainees can view these by clicking on the Awards icon.

(2)* Upgrade to the Trainer Web-Based Monitoring Software*. As participants are enrolled, an account is created on the trainer's monitoring website. Approximately every 24 hours the tablet attempts to connect with the server via the Internet to upload tablet usage data, providing the trainer with updated information on the number of minutes spent on various training components with each tablet session as well as the number of visits and time spent on each training activity. The data can be viewed at the website or downloaded in tabular form for further analysis.

(3)* Self-Contained Training Program*. While tablet functionality remains intact, the EPIC Wheels program is being operated as a stand-alone program with other applications hidden using a custom launcher program. All training content is included on the tablet and can be operated independent of Internet connectivity. A mobile WiFi device (AirCard 763S mobile hotspot, Sierra Wireless Inc, Richmond, BC) automatically connects the tablet to the Internet when it is in range (up to 34 metres indoors). The tablet can then update any voicemail messages between trainee and trainer as well as perform its daily upload of tablet training data. However, even if the tablet fails to connect through the hotspot to the Internet, it will continue to operate and record data independently until such time as the connection is reestablished.

(4)* Protection and Safety*. To protect trainee information, the program requires a password for access to protect trainee information and all data is encrypted before storage and uploaded to the secure server. A screen protector on the tablet reduces glare and protects the viewing surface from damage. The software requires trainees to complete the safety content section before permitting access to the remaining training content, and for higher risk content (e.g., popping casters) a pop-up window requires trainees to acknowledge compliance or click on a link to review the safety section. The BORG perceived rate of exertion scale was introduced in the safety section and trainees instructed to limit their effort to “somewhat hard” to prevent overexertion. In addition, content specific to care providers is provided including strategies to enhance effective training, safe spotting, and demonstration of techniques for assisting the wheelchair user during challenging or high-risk activities (e.g., high curbs and steps).

## 4. Discussion

We were successful in achieving the three objectives of this project. Our older adult MWC user partners were engaged throughout the design and implementation process and all stakeholder groups provided substantial contributions to the development of a clinical prototype that is currently being evaluated in a RCT. The PAD framework proved to be a valuable approach to creating the EPIC Wheels program. The iterative consultation process provided critical input into the evolving content and user interface. Incorporating a number of stakeholder groups provided validation for relevance and appropriateness of the included content. The MWC users confirmed the scope of skills included was comprehensive and contributed to inclusion of additional material such as the task of carrying an item while propelling a wheelchair. Care providers negotiated that training content around some high-level skills (e.g., wheelies and ascending steps) should be restructured with assisted, rather than independent, strategies. The clinician groups confirmed skills that were most enabling and often neglected among older adults, such as transient wheelies, and provided input on teaching strategies. Focus groups were particularly useful as they facilitated interaction and discussion among participants. The resulting dialogue was often animated and engaging, and there was not always agreement or consensus. While this made analysis more challenging, the outcome was a richer and more comprehensive product with greater potential for application to a broad audience.

The critiques and recommendations by stakeholders proved to be consistent with, and confirmatory of, the theoretical bases with which EPIC Wheels was created. Four key components of self-efficacy, as proposed in Social Cognitive Theory [53], were evident. The sequencing of skills from basic to advanced and the inclusion of multiple training activities for each skill graded from simple to complex maximize opportunity for successful skill performance or* mastery experience* which has the strongest influence on self-efficacy [54]. Early success experiences induce confidence that more difficult skills are attainable and enhance perseverance among trainees. Progress is monitored by trainers, who encourage skill advancement following successful performance but before proficiency, creating a “just right” challenge as proposed in the occupational therapy literature [55, 56]. The recommendation to include age-appropriate demonstrators of both sexes corresponds to* vicarious experience* or the observation of a comparable peer achieving success in a given skill, which is the penultimate factor influencing self-efficacy [54]. Knowles [29] also promotes the value of modeling to provide a rationale for older adults to pursue a specific skill, as it has been associated with improvement in skill performance [57]. A third component is the encouragement of meaningful others, or* verbal persuasion*. Stakeholders advocating for regular monitoring and follow-up by the trainer and for inclusion of spotting, training, and feedback strategies specifically for care providers in the EPIC Wheels program were particularly relevant in this regard. Finally, appropriate management and interpretation of one's* physiological state* is important to wheelchair confidence. The inclusion of games and other engaging training activities increase motivational investment while distracting trainees from the demands of performing mobility skills. While some older adults may be unfamiliar with computer games, we anticipate their inclusion will increase training time as recent studies show promising results in this regard, even among the very old [32, 33]. We also included information on self-monitoring physical expenditure, including information on the BORG Perceived Rate of Exertion scale [58] and parameters for not exceeding the recommended level of “somewhat hard” during training, based on best practice guidelines [59].

Stakeholders also provided input that aligned with Andragogical principles. Adult learners, particularly older adults, prefer an autonomous and self-directed approach that is goal-oriented and respectful [31]. The EPIC Wheels program allows trainees to control the time and location of training activity and provides continuous updates on the number of components completed and total time spent in practice. Flexible navigation ensures trainees can control which specific skills they want to work on, advancing when they feel ready and revisiting material if desired. Trainers assist in prioritizing skills most relevant to trainee goals and activities of interest. Providing a rationale for each skill in relation to specific occupations of interest, inclusion of typical daily activities and commonplace equipment for practice, and demonstration of incorrect performance with the resultant hazards offer a practical and life-experience approach to learning consistent with Andragogical principles.

A key benefit of the PAD approach was optimizing the tablet interface. Despite the numerous benefits that a tablet offers, such as touch screen access, interactivity, portability, and Internet connectivity, acceptance and adherence by older adults are critical to the success of this home program. By bringing evolving prototypes back to the target users, as well as other stakeholders, we were able to ensure usability by older adults. Although older adults are less likely to use technologies such as computers and cell phones than young people, computer use is continuing to grow. Recent studies in the United States found 84% of those over 60 years had experience with computers [60] and 40% of those over 65 years are regular computer and Internet users [61]. Use of a computer tablet involves some new learning, and age-related declines in memory and fluid intelligence may restrict uptake. These issues can be addressed through self-paced training structured for success experiences to build confidence and adapting the interface design for familiarity and ease of use with minimal memory requirements [57, 62].

At the conclusion of the PAD process the EPIC Wheels program and training tablet demonstrated robust and consistent performance and are currently being evaluated in a randomized controlled trial with novice older adult MWC users [52]. The training program is downloaded onto a tablet with an individualized identity and password for each trainee and a corresponding identity is created on the trainer's website, located on a secure server. The wheelchair user can perform training independently or with supervision by a care provider, particularly when more advanced or higher risk skills are being learned.

Future development will focus on several improvements. Communication between trainee and trainer is currently conducted via voicemail, but the capacity for recorded video communication is already in place. Expediting video data transfer and integration of a video player applet are under development and the potential for real-time video communication is also being explored. The software content and user interface are self-contained and preloaded on the trainee tablet. A content management software program will provide the potential for trainers to customize a trainee's program, adding and removing content as desired. In the future, this would allow a trainer to release new content over time via the Internet. Using built-in or external sensors could expand the scope of interactive training activities used and collection of performance-based data.

Some limitations with the EPIC Wheels program should be noted. The training content specifically targets MWC users who propel with both upper extremities, including those who also use one or both lower extremities. Other propulsion strategies, such as one arm and one leg used by individuals with hemiplegia, and mobility equipment (e.g., power wheelchairs, scooters) are common and require a different set of skills. Such content will need to be created to address these users groups. The software developers were proficient with the Android platform and EPIC Wheels is currently available only on these devices; creating a version compatible with the Apple iPad would facilitate broader appeal and availability. Finally, while the tablet tracks all program interactions and uploads detailed activity information to the trainer website, there is no way to verify that trainees physically engage in training beyond viewing the program content. In future, synchronizing training activities with input from a data logger or tablet accelerometer may address this issue.

## 5. Conclusion

A participatory action design process proved valuable in the development and refinement of a tablet-based wheelchair skills home training program. The involvement of older adult wheelchair users, as well as care providers and clinician stakeholders, was critical to achieving a product that was both comprehensive and acceptable to the target users. The contributions of these research partners confirmed the underlying theoretical principles of self-efficacy and adult learning theory upon which the program was developed. The clinical prototype that emerged is currently under evaluation in a randomized controlled trial and further enhancements to the current program are anticipated in the near future.

## Supplementary Material

The supplementary material includes a copy of the Training Progress Sheet. Participants receive a print copy of this handout in their user guide. All training content is outlined in sequence and broken down into sections that correspond to the EPIC Wheels software interface. This sheet allows participants to locate specific content easily and visually track progress through the entire program. At each training session, the trainer can highlight specific content for the participant to work on and make written comments for future reference.
