# Supplementary material for: Development of a Wheelchair Skills Home Program for Older Adults Using a Participatory Action Design Approach
Source: Biomed Res Int. 2014 Sep 4;2014:172434. doi: 10.1155/2014/172434 (PMC4167954; doi:10.1155/2014/172434)
Supplement: Supplementary file 1 — The supplementary material includes a copy of the Training Progress Sheet. Participants receive a print copy of this handout in their user guide. All training content is outlined in sequence and broken down into sections that correspond to the EPIC Wheels software interface. This sheet allows participants to locate specific content easily and visually track progress through the entire program. At each training session, the trainer can highlight specific content for the participant to work on and make written comments for future reference. [file 172434.f1.docx]

**Training Progress Sheet**

| **Safety** | Equipment | |  |
| --- | --- | --- | --- |
|  | Supervision & Spotting | |  |
|  | Spotter Information | |  |
|  | Tipping and Falling | |  |
|  | Spotter’s strap | |  |
|  | Types of Injuries | |  |
|  |  |  |  |
| **Components** | Wheelchair Parts | *Wheel Locks* | Wheel Locks |
|  |  |  | Activity 1 |
|  |  |  | Activity 2 |
|  |  | *Footrests* | Footrests |
|  |  |  | Activity 1 |
|  |  |  | Activity 2 |
|  |  | *Folding mechanism* | Folding Mechanism |
|  |  |  | Activity 1 |
|  |  |  | Activity 2 |
|  |  | *Anti-tippers* | Anti-tippers |
|  |  |  | Activity 1 |
|  |  |  | Activity 2 |
|  |  | *Drive Wheels* | Drive Wheels |
|  |  | *Casters* | Casters |
|  |  |  | Activity 1 |
|  |  |  | Activity 2 |
|  |  |  | Activity 3 |
|  |  |  | Activity 4 |
|  |  | *Arm Rests* | Arm Rests |
|  |  |  | Activity 1 |
|  | Body Position | *Body Position* | Body Position |
|  |  |  | Activity 1 |
|  |  |  | Activity 2 |
|  |  | *Weight Shift* | Weight Shift |
|  |  |  | Activity 1 |

| **Propelling** | *Basic Propulsion* | Hand Position | |  |
| --- | --- | --- | --- | --- |
|  |  | Activity 1 | |  |
|  |  | Activity 2A | |  |
|  |  | Activity 2B | |  |
|  |  | Activity 2C | |  |
|  |  | Activity 2D | |  |
|  |  | Activity 3 | |  |
|  | *Pushing Techniques* | Pushing Techniques | |  |
|  | *Coasting* | Coasting | |  |
|  |  | Activity 1 | |  |
|  |  | Activity 2 | |  |
|  |  |  | |  |
| **Skills Section A** | Propelling Forwards | *Propelling Forwards* | | Propelling |
|  |  |  | | Activity 1 |
|  |  |  | | Activity 2 |
|  |  | *Making Corrections 1* | | Corrections 1 |
|  |  |  | | Activity 1 |
|  |  | *Making Corrections 2* | | Corrections 2 |
|  |  |  | | Activity 1 |
|  | Propelling Backwards |  | Propelling Backwards | |
|  |  |  | Activity 1 | |
|  | Turns | *Turning* | | Turning |
|  |  |  | | Activity 1 |
|  |  |  | | Activity 2 |
|  |  |  | | Activity 3 |
|  |  | *Spin Turns* | | Spin Turns |
|  |  |  | | Activity 1 |
|  |  |  | | Activity 2 |
|  |  |  | | Activity 3 |
|  |  | *Quick Spin Turns* | | Quick Spin Turns |
|  |  |  | | Activity 1 |
|  |  |  | | Activity 2 |
|  |  |  | | Activity 3 |
|  |  | *Moving Turns 1* | | Moving Turns 1 |
|  |  |  | | Activity 1 |
|  |  |  | | Activity 2 |

|  | Turns (con’t) | *Moving Turns 2* | Moving Turns 2 |
| --- | --- | --- | --- |
|  |  |  | Activity 1 |
|  |  |  | Activity 2 |
|  |  |  | Activity 3 |
|  |  |  | Activity 4 |
|  |  | *Narrow Spaces* | Narrow Spaces |
|  |  |  | Activity 1 |
|  |  |  | Activity 2 |
|  |  | *Backward Turns* | Backward Turns |
|  |  |  | Activity 1 |
|  |  |  | Activity 2 |
|  |  |  | Activity 3 |
|  |  | *Moving Sideways* | Moving Sideways |
|  |  |  | Activity 1 |
|  |  |  | Activity 2 |
|  | Avoiding Obstacles | *Avoiding Obstacles* |  |
|  |  | *Activity 1* |  |
|  |  | *Activity 2* |  |
|  |  | *Activity 3* |  |
|  |  | *Activity 4* |  |
|  |  | *Activity 5* |  |
|  | Reaching | *Reaching Sideways* | Sideways |
|  |  |  | Activity 1 |
|  |  | *Reaching Down* | Down |
|  |  |  | Activity 1 |
|  |  | *Reaching Up* | Up |
|  |  |  | Activity 1 |
|  | Carrying Objects | *Carrying Objects 1* |  |
|  |  | *Carrying Objects 2* |  |
|  |  | *Carrying Objects 3* |  |
|  |  | *Carrying Objects 4* |  |
|  |  | *Carrying Objects 5* |  |
|  |  | *Carrying Objects 6* |  |
|  |  | *Carrying Objects 7* |  |

| **Skills Section B** | Popping Casters | *Self* | Popping Casters |
| --- | --- | --- | --- |
|  |  |  | Activity 1 |
|  |  |  | Activity 2 |
|  |  |  | Activity 3 |
|  |  |  | Activity 4 |
|  |  |  | Activity 5 |
|  |  | *Assisted* | Popping Casters |
|  | Small Obstacles | *Forwards* | Forwards |
|  |  |  | Activity 1 |
|  |  |  | Activity 2 |
|  |  |  | Activity 3 |
|  |  |  | Activity 4 |
|  |  | *Backwards* | Backwards |
|  |  |  | Activity 1 |
|  |  | *Foot-Assisted* | Foot-Assisted |
|  |  |  | Activity 1 |
|  |  |  | Activity 2 |
|  |  | *Momentum* | Momentum |
|  |  |  | Activity 1 |
|  |  |  | Activity 2 |
|  |  | *Assisted* | Assisted |
|  | Gaps | *Forwards* | Forwards |
|  |  | *Backwards* | Backwards |
|  |  |  | Activity 1 |
|  |  |  | Activity 2 |
|  |  |  | Activity 3 |
|  |  | *Assisted* | Assisted |
|  | Soft Surfaces | *Carpet* | Carpet |
|  |  | *Grass* | Grass |
|  |  | *Snow* | Snow |
|  |  | *Activities* | Activity 1A |
|  |  |  | Activity 1B |
|  |  |  | Activity 2 |
|  |  |  | Activity 3 |
|  |  |  | Activity 4 |
|  |  | *Assisted* | Assisted |

|  | Shallow Inclines | *Inclines - Up* | Forwards |
| --- | --- | --- | --- |
|  |  |  | Activity 1 |
|  |  |  | Backwards |
|  |  | *Inclines - Down* | Inclines Down |
|  |  |  | Activity 1 |
|  |  |  | Activity 2 |
|  |  | *Assisted* | Assisted |
|  | Side Slopes | *Side Slopes* |  |
|  |  | *Activity 1* |  |
|  | Doors | *Doors* |  |
|  |  | *Activity 1* |  |
|  |  | *Activity 2* |  |
|  | Steep Inclines | *Inclines Up* | Inclines Up |
|  |  |  | Activity 1 |
|  |  | *Inclines Down* | Inclines Down |
|  |  |  | Activity 1 |
|  |  |  | Activity 2 |
|  | High Obstacles | *Up with Assist* |  |
|  |  | *Up without Assist* |  |
|  |  | *Down with Assist* |  |
|  |  | *Down without Assist* |  |
|  | Steps & Stairs | *Steps/Stairs with Assist* | |
